# Preventive Effect of Small-Leaved Kuding Tea (*Ligustrum robustum* (Roxb.) Bl.) Polyphenols on D-Galactose-Induced Oxidative Stress and Aging in Mice

**DOI:** 10.1155/2019/3152324

**Published:** 2019-05-22

**Authors:** Bihui Liu, Ruidong Ma, Jing Zhang, Peng Sun, Ruokun Yi, Xin Zhao

**Affiliations:** ^1^Chongqing Collaborative Innovation Center for Functional Food, Chongqing University of Education, Chongqing 400067, China; ^2^Chongqing Engineering Research Center of Functional Food, Chongqing University of Education, Chongqing 400067, China; ^3^Chongqing Engineering Laboratory for Research and Development of Functional Food, Chongqing University of Education, Chongqing 400067, China; ^4^College of Biological and Chemical Engineering, Chongqing University of Education, Chongqing 400067, China; ^5^Department of Cardiothoracic Surgery, the First Affiliated Hospital of Chengdu Medical College, Chengdu 610500, China; ^6^Environment and Quality Inspection College, Chongqing Chemical Industry Vocational College, Chongqing 401228, China

## Abstract

Small-leaved Kuding tea is a traditional Chinese tea that is rich in polyphenols. In the current study, we investigated the preventive effect of small-leaved Kuding tea (SLKDT) on D-galactose-induced oxidative aging in mice. Changes in serum, skin, liver, and spleen of experimental animals were determined using biochemical and molecular biology techniques. Biochemical analysis demonstrated that polyphenol extract of SLKDT (PSLKDT) improved the indices of the thymus, brain, heart, liver, spleen, and kidney function in model mice. PSLKDT prevented a decrease in the levels of superoxide dismutase (SOD), glutathione peroxidase (GSH-Px), and glutathione (GSH) as well as an increase in nitric oxide (NO) and malondialdehyde (MDA) levels in serum, liver, and spleen. Pathological assessment also showed that PSLKDT reduced oxidative damage induced by D-galactose in skin, liver, and spleen. We further found that PSLKDT upregulated neuronal nitric oxide synthase (nNOS), endothelial nitric oxide synthase (eNOS), Cu/Zn-SOD, Mn-SOD, catalase (CAT), heme oxygenase-1 (HO-1), nuclear factor (nuclear factor-erythroid 2 related factor 2 (Nrf2), *γ*-glutamylcysteine synthetase (*γ*-GCS), and NAD(P)H dehydrogenase [quinone] 1 (NQO1) mRNA expression and downregulated inducible nitric oxide synthase (iNOS) mRNA expression. Protein levels of SOD1 (Cu/Zn-SOD), SOD2 (Mn-SOD), CAT, GSH1 (*γ*-glutamate-cysteine ligase), and GSH2 (glutathione synthetase) in the liver and spleen were also increased by PSLKDT treatment. Collectively, these results indicate that PSLKDT is effective in preventing D-galactose-induced oxidative aging in mice, and its efficacy is significantly higher than antioxidant vitamin C. Because PSLKDT is a potent antioxidant and antiaging polyphenol, Kuding tea rich in PSLKDT should be considered an ideal drink with antioxidative and antiaging effects.

## 1. Introduction

Kuding tea, the leaf of broadleaf holly, has been used as herbal tea in China for more than 2,000 years. Its production and consumption are second only to regular tea (*Camellia* tea); Kuding tea is viewed as the best among the non-*Camellia* teas [1]. Based on the different plant origins, Kuding tea can be divided into big-leaved and small-leaved Kuding tea (SLKDT). Big-leaved Kuding tea is derived from the plant* I. kudingcha* C. J. Tseng, whereas small-leaved Kuding tea originated from the plant* Ligustrum robustum* (Roxb.) Bl. [2]. Studies have indicated that SLKDT has a variety of medicinal properties such as dispersing heat and resolving toxins, anti-inflammatory, bactericidal, improving hypertension and hyperlipidemia, helping in weight control, and diuretic [3]. SLKDT contains various bioactive components, including polyphenols, flavonoids, amino acids, caffeine, essential oils, and trace elements [4]. In particular, the polyphenol content in SLKDT is as high as 6% [5], and thus, SLKDT drink is viewed as a health product.

Aging is a natural, gradual process occurring in organisms, which involves incurring various damage to the body. Aging is associated with many diseases, including hypertension, type-2 diabetes, atherosclerosis, and senile dementia [6–8]. Excessive production of reactive oxygen species can damage large biological molecules that lead to cell senescence. During aging, increased production of hydrogen peroxide by the mitochondria can cause oxidative damage to the body [9]. How to achieve a balanced oxidative and reductive process (redox homeostasis) is thus a key issue in aging research. In addition, the identification of regulatory genes involved in redox homeostasis has become a new approach to delay the aging process [10]. D-galactose is an agent often used for experimental induction of aging, and animal aging models induced with D-galactose are similar to natural aging. The consumption of a small amount of D-galactose can be converted to glucose in the body, which in turn participates in metabolism. However, intake of large amounts of galactose causes metabolic disorder in cells, resulting in alterations in the activity of oxidative enzymes, and increased production of superoxide anions and oxidative products, which cause oxidative damage to the structure and function of large biological molecules, ultimately leading to aging of the body [11]. Aging models established with the administration of D-galactose have been widely used to assess the antiaging efficacy of antioxidant compounds and develop the antiaging heath products. It has been shown that structural characters of plant polyphenols afford them potent antioxidant and free radical-scavenging capabilities. Phenolic hydroxyl groups, particularly the* ortho*-phenolic hydroxyl groups in pyrocatechin and pyrogallol, are easily oxidized to quinones, which quench reactive oxygen species and inhibit the generation of lipid free radicals, resulting in reduced oxidation in tissues [12]. Plant polyphenols have been proven to have antioxidant activity in both animal and human studies [13, 14]. For example, green tea polyphenols have been shown to impart antioxidant and antiaging effects by enhancing three major antioxidant enzymes, namely, SOD, GSH-PX, and CAT [15].

In this study, we administered polyphenol extract of SLKDT (PSLKDT) made from SLKDT to D-galactose-treated mice (oxidative aging model) and determined its effect on relevant serum and tissue markers as well as oxidative aging-related genes for mechanistic analysis. The results provide useful information for conducting future human studies and in developing health-promoting products.

## 2. Materials and Methods

### 2.1. Extraction of PSLKDT

Five hundred grams of SLKDT was made into powder and dissolved in 50 mL of 45% (w/w) ethanol solution. The mixture was extracted under 90°C for 30 min twice, and then two parts of the extract were combined. After the extract was adjusted to pH 6.0, 800 mL of precipitation solution containing 30 g AlCl_3_ and 60 g ZnCl_2_ was added to the extract for precipitation, followed by centrifugation at 3,000 rpm for 10 min. Next, 1,000 mL of 12% HCl was added to the precipitated extract for transfer. After that, the supernatant was collected and mixed with 100 mL of ethyl acetate for further extraction twice. The resulting extraction solution was dried under rotating evaporation to generate PSLKDT [16].

### 2.2. Analysis of PSLKDT Composition

Catechin (0.9 mg), caffeic acid (2.0 mg), GCG (1.9 mg), ferulic acid (1.2 mg), isochlorogenic acid B (2.0 mg), and isochlorogenic acid A (1.1 mg) were separately added to each EP tube containing 2 mL of methanol. The prepared standard stock solutions contained 0.45 mg/mL catechin, 1.0 mg/mL caffeic acid, 0.95 mg/mL GCG, 0.6 mg/mL ferulic acid, 1.0 mg/mL isochlorogenic acid B, and 0.55 mg/mL isochlorogenic acid A, respectively. A mixed standard solution was prepared by mixing 100 *μ*L of each standard stock solution together in a vial. Liquid chromatography (UltiMate3000 HPLC System, Thermo Fisher Scientific, Waltham, MA, USA) conditions were Thermo Accucore C18 column (150 × 1.6 mm, 2.6 *μ*m); temperature: 30°C, high limit 110.0°C, and low limit 5.0°C; flow rate: 0.6 mL/min; detector: UV detector; detection wavelength: 280 nm; mobile phase: acetonitrile and 0.1% formic acid; gradient elution with constant flow rate and altered ratios of acetonitrile: 0.1% formic acid with time as shown in Table 1; sample injection: 2 *μ*L/injection × 6 times; standard mixture (6 standards) was injected in 21–25 *μ*L gradient, for a total of 5 times.

### 2.3. Experiments Involving an Oxidative Aging Animal Model

A total of 50 specific pathogen-free male and female ICR mice (six-week-old) were acclimated for one week and then randomly divided to five groups (n=10/group, half for each gender): normal control, model, low-dose PSLKDT gavage (PSLKDT-L), high-dose PSLKDT gavage (PSLKDT-H), and vitamin C gavage (Vc); the mice in normal control and model groups did not treatment; the mice in PSLKDT-L, PSLKDT-H, and Vc group were treated with 50 mg/kg PSLKDT, 100 mg/kg PSLKDT, and 100 mg/kg vitamin C every day by gavage, respectively. After a four-week gavage treatment, all mice except for those in the normal control group were intraperitoneally injected with D-galactose (120 mg/kg, daily) for six weeks, whereas all gavage treatments continued. After six weeks, all mice were fasted for 24 h and then euthanized as previously described [17]. Blood was collected via heart puncture, and thymus, brain, heart, liver, spleen, and kidney weights were recorded for organ index calculation: Organ index = Organ weight (g)/Mouse body weight (g) × 100. This study was conducted in accordance with the Declaration of Helsinki, and the protocol was approved by the Ethics Committee of Chongqing Collaborative Innovation Center for Functional Food (201807002B).

### 2.4. Measurements of NO and MDA Levels and SOD and GSH-Px Activity in Serum and Liver

Serum was obtained by centrifuging mouse blood at 4,000 rpm for 10 min. Serum NO and MDA levels and SOD and GSH-Px activity were analyzed using ELISA kits following the manufacturers' instructions. Liver was homogenized and centrifuged to obtain the supernatant, which was used for measuring NO and MDA levels and SOD and GSH-Px activity using the same methods (Nanjing Jiancheng Bioengineering Institute, Nanjing City, China) as mentioned above for serum samples.

### 2.5. Histology Analysis of Skin, Liver, and Spleen

A piece (about 0.5 cm^2^) of skin, liver, or spleen was fixed in 10% formalin solution for 48 h, which was followed by the processes of dehydration, paraffin embedding, sectioning, and H&E staining. Tissue histology was examined under an optical microscope (BX43 microscope, Olympus, Tokyo, Japan).

### 2.6. Quantitative PCR (qPCR) Assay

Skin, liver, and spleen tissues were homogenized and total RNA was extracted with RNAzol (Thermo Fisher Scientific). Extracted total RNA was diluted to 1 *μ*g/*μ*L, and 5 *μ*L was used for reverse transcription to synthesize cDNA. Two microliters of cDNA was mixed with 10 *μ*L SYBR Green PCR Master Mix and 1 *μ*L of each primer (Thermo Fisher Scientific, Table 2) for PCR reaction under the following conditions: 95°C for 60 s, followed by 40 cycles of 95°C for 15 s, 55°C for 30 s, and 72°C for 35 s, and a final 95°C for 30 s, followed by 55°C for 35 s. GAPDH was used as internal reference, and relative gene transcript levels were calculated using 2^−ΔΔCt^ method [18].

### 2.7. Western Blot Analysis

A piece (100 mg) of skin, liver, or spleen tissue sample was homogenized in 1 mL of RIPA solution containing 10 *μ*L of PMSF, and the supernatant was obtained after centrifugation at 12,000 rpm, 4°C, for 5 min. Total protein in supernatant was determined using the BCA protein quantitative kit. All samples were diluted to a concentration of 50 *μ*g/mL and then mixed with sample buffer at a ratio of 4:1. After the samples were denatured at 100°C for 5 min, these were electrophoresed in a SDS-polyacrylamide gel for 50 min and then transferred to a methanol-activated PVDF membrane. After blocking with 5% nonfat dry milk in TBST for 1 h, the membranes were incubated with the respective primary antibodies at 25°C for 2 h. The membranes were rinsed and then incubated with the corresponding secondary antibodies at 25°C for 1 h. After the membrane was incubated in Supersignal West Pico PLUS, all bands were quantified by an iBright FL1000 (Thermo Fisher Scientific) [19].

### 2.8. Statistical Analysis

All data were analyzed using SAS9.1 statistical software (SAS Institute, Cary, NC, USA), and the results were presented as means of three independent experiments. The differences among groups were assessed using one-way ANOVA with the Dunnett's test for post hoc analysis, and significance was set at* p* < 0.05 [19].

## 3. Results

### 3.1. PSLKDT Composition

**Figure 1:**
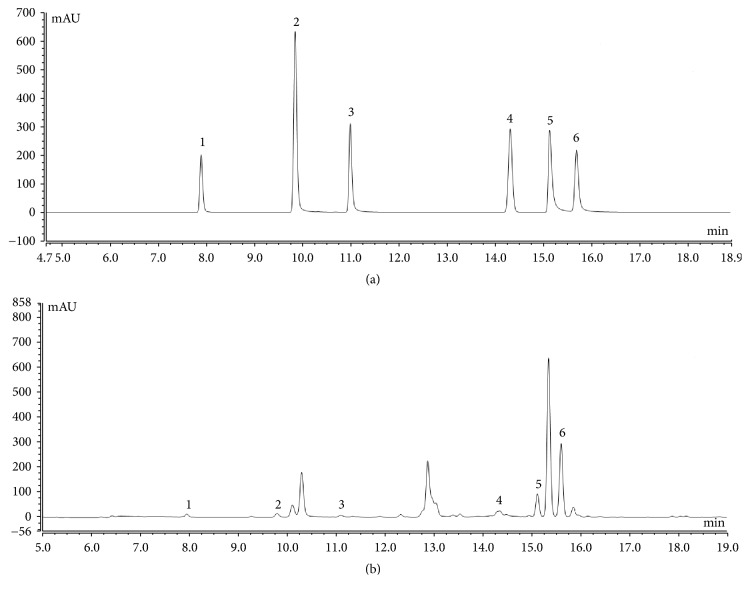
Polyphenol constituents of small-leaved Kuding tea. (a) Standard chromatograms; (b) Polyphenols of small-leaved Kuding tea chromatograms. 1: catechin; 2: caffeic acid; 3: gallocatechin gallate (GCG); 4: ferulic acid; 5: isochlorogenic acid B; 6; isochlorogenic acid A.

HPLC analysis revealed that PSLKDT contained six types of polyphenols, including catechin, caffeic acid, GCG, ferulic acid, isochlorogenic acid B, and isochlorogenic acid A (Figure 1). Isochlorogenic acid B and isochlorogenic acid A content, which were higher than catechin, caffeic acid, GCG, ferulic acid, were the primary bioactive components in PSLKDT (Table 3).

### 3.2. Organ Indices

The organ indices of thymus, brain, heart, liver, spleen, and kidney were highest in mice from the normal control group and lowest in the model group (Table 4). While both PSLKDT and VC increased organ indices, the efficacy of the former was greater than that of the latter. These results indicate that PSLKDT-induced reduction in organ indices may reflect prevention of the body tissue atrophy.

### 3.3. NO, SOD, GSH-Px, GSH, and MDA Levels

SOD, GSH-Px, and GSH activities in the serum, liver, and spleen were highest in mice from the normal control group and lowest in the model group (Tables 5, 6, and 7). PSLKDT treatment increased SOD, GSH-Px, and GSH activities (*p*<0.05) and decreased NO and MDA levels (*p*<0.05). PSLKDT was more effective than VC in terms of these effects. The values of the above parameters in the PSLKDT group were roughly similar to those of the control group.

### 3.4. Skin, Liver, and Spleen Pathology

**Figure 2:**
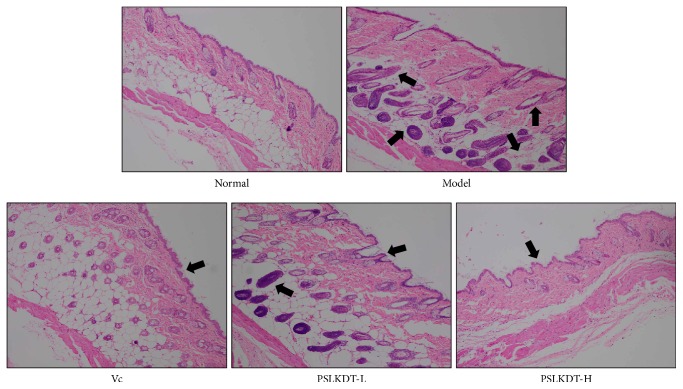
H&E pathological observation of skin in mice. Magnification 100×. Vc: mice treated with vitamin C (100 mg/kg); PSLKDT-L: mice treated with low concentration of polyphenol of small-leaved Kuding tea (50 mg/kg); PSLKDT-H: mice treated with high concentration of polyphenol of small-leaved Kuding tea (100 mg/kg).

In the normal group, the epidermis of skin tissue was complete and thin, and the dermal fibrous tissue was evenly distributed (Figure 2). In the model group, the epidermis tissue thickness was not uniform, the structure was incomplete, the dermis fibrous tissue was loosely distributed, there were often ruptures, and more inflammatory cells infiltrated. Both Vc and PSLKDT could alleviate the oxidative damage caused by D-galactose to the skin tissue of mice, and the effect of PSLKDT-H was better than that of PSLKDT-L and Vc, which could make the skin tissue close to the normal skin of mice.

**Figure 3:**
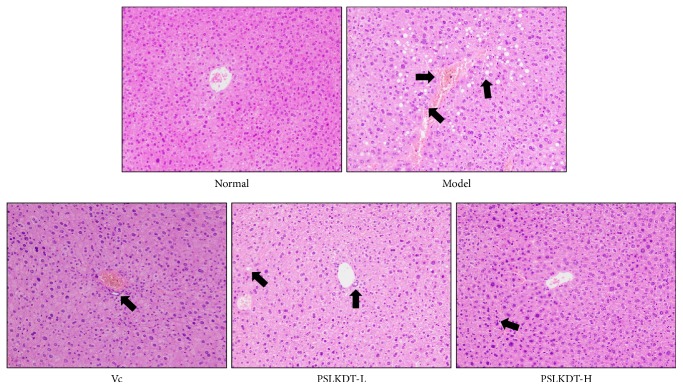
H&E pathological observation of liver in mice. Magnification 100×. Vc: mice treated with vitamin C (100 mg/kg); PSLKDT-L: mice treated with low concentration of polyphenol of small-leaved Kuding tea (50 mg/kg); PSLKDT-H: mice treated with high concentration of polyphenol of small-leaved Kuding tea (100 mg/kg).

In the normal group, the liver tissue structure of mice was intact, the hepatocytes were radially arranged around the central vein, the hepatic lobules were clearly defined, the hepatocytes were clearly demarcated, and the cytoplasm was abundant (Figure 3). In the model group, the hepatic tissue structure was incomplete, the hepatic lobule boundary was not clear, the central vein was not clear, and there were obvious fat vacuoles. After the treatment with Vc and PSLKDT-H, no obvious fat vacuoles were found in the liver tissue of D-galactose induced oxidation mice, and the hepatic lobule contour and hepatocyte boundary were relatively clear. In addition, the hepatic cells in the liver tissue of PSLKDT-H treated mice were also radially arranged around the central vein. There were also individual fat vacuoles in the liver tissues of PSLKDT-L treated mice, and the hepatic cell structure was partially incomplete.

**Figure 4:**
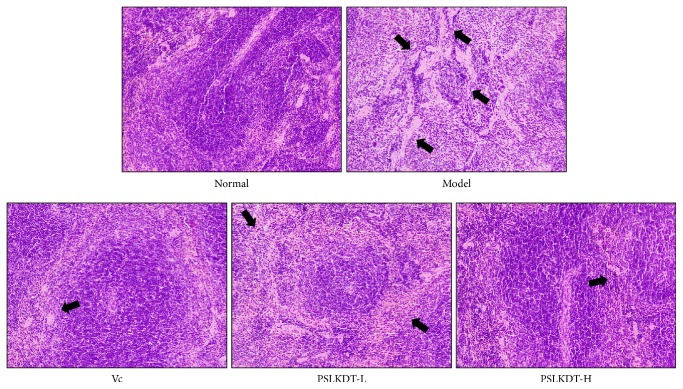
H&E pathological observation of spleen in mice. Magnification 100×. Vc: mice treated with vitamin C (100 mg/kg); PSLKDT-L: mice treated with low concentration of polyphenol of small-leaved Kuding tea (50 mg/kg); PSLKDT-H: mice treated with high concentration of polyphenol of small-leaved Kuding tea (100 mg/kg).

Figure 4 shows that the spleen in normal control mice exhibited clear and intact structures, corticomedullary junctions were clear, and the cells were orderly arranged. In contrast, the spleen of the model mice showed abnormal shapes and disordered structures, enlarged red pulp blood sinuses filled with large number of red blood cells, and reduced white pulp lymphocytes, narrow red pulp cords, and less dense arrays of cells. All these D-galactose-induced histologic changes in the spleen were reduced by PSLKDT treatment to a level close to the normal condition. These histological observations in the skin, liver, and spleen indicate that PSLKDT may exert protective effects in oxidative aging-associated tissue damage, and this effect is superior to VC.

### 3.5. mRNA Expression of Redox-Related Markers in Liver

**Figure 5:**
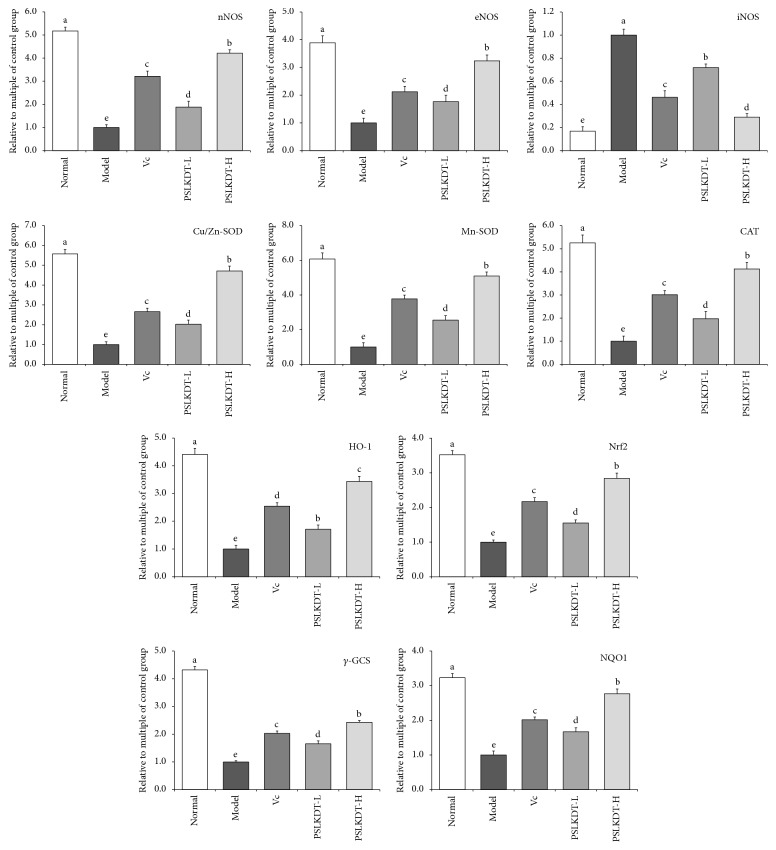
The mRNA expression in liver of mice. ^a-e^Mean values with different letters in the bar are significantly different (p< 0.05) according to Duncan's multiple-range test. Vc: mice treated with vitamin C (100 mg/kg); PSLKDT-L: mice treated with low concentration of polyphenol of small-leaved Kuding tea (50 mg/kg); PSLKDT-H: mice treated with high concentration of polyphenol of small-leaved Kuding tea (100 mg/kg).

mRNA expression of nNOS, eNOS, Cu/Zn-SOD, Mn-SOD, CAT, HO-1, Nrf2, *γ*-GCS, and NQO1 in liver of normal control mice was higher (p<0.05), and iNOS was lower (p<0.05) than mice in all the other groups (Figure 5). In D-galactose-induced oxidative aging model mice, the liver mRNA expression levels of nNOS, eNOS, Cu/Zn-SOD, Mn-SOD, CAT, HO-1, Nrf2, *γ*-GCS, and NQO1 significantly decreased, whereas iNOS significantly increased. All these D-galactose-induced changes were prevented by PSLKDT treatment (p<0.05), and PSLKDT was more effective than VC in this regard.

### 3.6. mRNA Expression of Redox-Related Markers in Spleen

**Figure 6:**
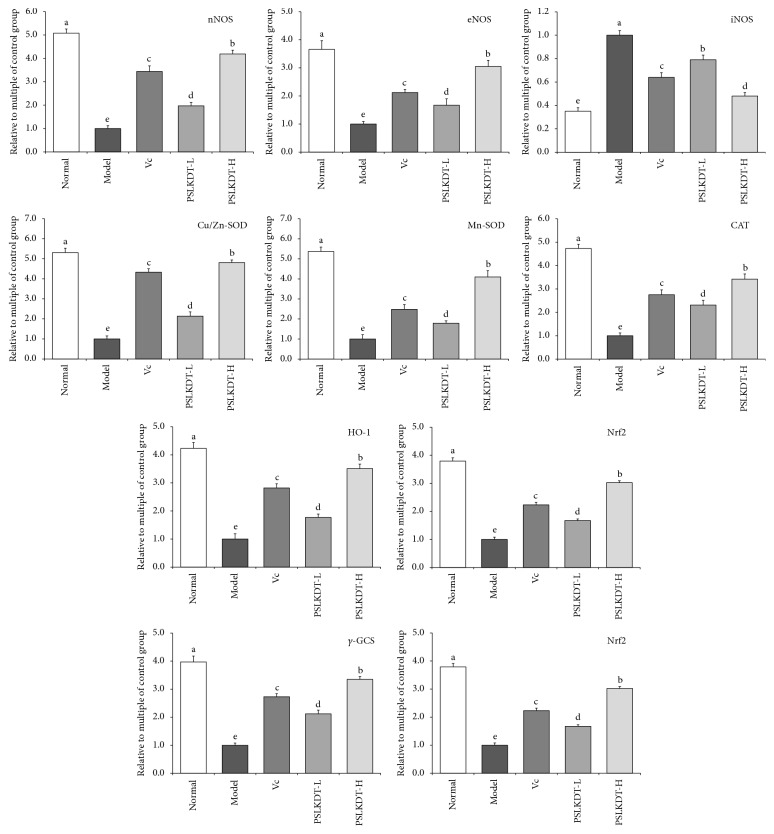
The protein expression in spleen of mice. ^a-e^Mean values with different letters in the bar are significantly different (p< 0.05) according to Duncan's multiple-range test. Vc: mice treated with vitamin C (100 mg/kg); PSLKDT-L: mice treated with low concentration of polyphenol of small-leaved Kuding tea (50 mg/kg); PSLKDT-H: mice treated with high concentration of polyphenol of small-leaved Kuding tea (100 mg/kg).

Figure 6 shows that mRNA expression of the above-mentioned redox markers in the spleen showed the same trends as that in the liver of mice among all the groups; i.e., mRNA expression of nNOS, eNOS, Cu/Zn-SOD, Mn-SOD, CAT, HO-1, Nrf2, *γ*-GCS, and NQO1 was highest, and iNOS was lowest in the spleen of normal control mice. Likewise, mRNA expression of these markers in the spleen of the model mice showed changes that were opposite to those in the control mice, i.e., the lowest mRNA expression of nNOS, eNOS, Cu/Zn-SOD, Mn-SOD, CAT, HO-1, Nrf2, *γ*-GCS, and NQO1, and highest mRNA expression of iNOS. PSLKDT and VC prevented these changes in model mice (p<0.05), and in the high-dose of PSLKDT group, mRNA expression of these markers was similar to that of normal control mice.

### 3.7. Protein Expression of Redox-Related Markers in Liver

**Figure 7:**
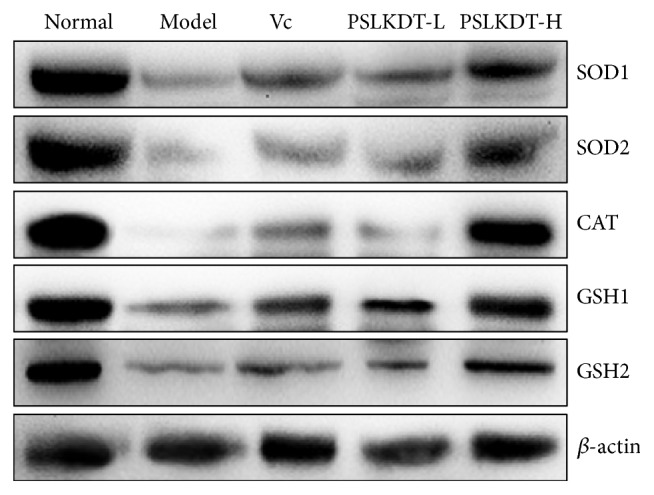
The protein expression in liver of mice. ^a-e^Mean values with different letters in the bar are significantly different (p< 0.05) according to Duncan's multiple-range test. Vc: mice treated with vitamin C (100 mg/kg); PSLKDT-L: mice treated with low concentration of polyphenol of small-leaved Kuding tea (50 mg/kg); PSLKDT-H: mice treated with high concentration of polyphenol of small-leaved Kuding tea (100 mg/kg).

Protein levels of redox-related markers in the liver were determined by western blotting (Figure 7). The results showed that SOD1, SOD2, CAT, GSH1, and GSH2 were lowest in model group mice and highest in normal control mice. The protein levels of these markers were upregulated by PSLKDT treatment, and this effect was more pronounced with PSLKDT than VC treatment.

### 3.8. Protein Expression of Redox-Related Markers in Spleen

**Figure 8:**
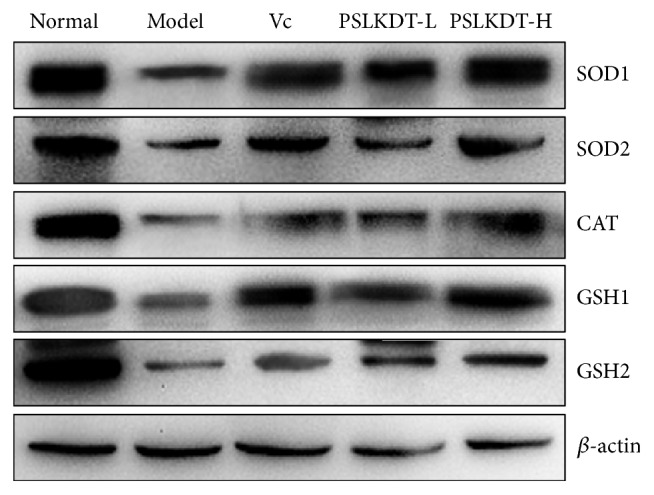
The protein expression in spleen of mice. ^a-e^Mean values with different letters in the bar are significantly different (p< 0.05) according to Duncan's multiple-range test. Vc: mice treated with vitamin C (100 mg/kg); PSLKDT-L: mice treated with low concentration of polyphenol of small-leaved Kuding tea (50 mg/kg); PSLKDT-H: mice treated with high concentration of polyphenol of small-leaved Kuding tea (100 mg/kg).

The protein expression levels of the above-mentioned redox markers in the spleen were similar to that in the liver as earlier described (Figure 8). SOD1, SOD2, CAT, GSH1, and GSH2 protein levels were higher in the spleen of normal control mice compared to all the other groups (p<0.05). The protein expression levels of these markers in the spleen of the high-dose PSLKDT-treated mice were similar to those of normal control mice and significantly different from those of mice treated with low-dose PSLKDT or VC. Protein expression levels of all these markers in the spleen of model mice were lower than all the other groups. These results indicate that PSLKDT can also upregulate protein expression of these markers in the spleen.

## 4. Discussion

Organ weight and organ index are indicators for assessing animal health condition, and changes in these indicators can reflect the aging status. When animals age, their thymus and brain undergo atrophy more strikingly than other organs; an aged thymus is a primary driver of immunosenescence, and an aged brain is a characteristic of systemic senescence [20]. Liver and kidney are the primary metabolic organs in mice and thus decreased weight and organ indices for these organs have direct impact on the body's metabolic functions. Because liver is also considered an immune organ in animals, altered indices in the liver affect the body's immune function [21]. Spleen as a primary component of the immune system plays an important role in the body's immune responses. Reduced spleen weight indicates spleen atrophy, which causes impaired immune function. Thus, measurement of animal's spleen index reflects the organ's structural and functional changes and thus serves as a valuable reference to determine whether an aging mouse model has been successfully established [22]. The results of the current study demonstrated that D-galactose treatment successfully induces oxidative aging in mice, resulting in reduction of organ index, and PSLKDT prevents D-galactose-induced reduction of organ index, which suggests an antiaging effect.

An increase in the release of excitatory amino acids such as glutamine can activate NMDA receptors, leading to Ca^2+^ influx. Elevated intracellular Ca^2+^ levels activate NOS to increase NO production. NO can increase Ca^2+^ influx to cause Ca^2+^ overload in cells, which directly inhibits mitochondrial energy generation. However, this process triggers production of O_2_^−^ and ·OH, leading to brain cell damage. NO can react with O_2_^−^ to generate peroxynitrite, which is decomposed to generate OH^−^ and NO_2_^−^ that promote cell death [23]. Repeated infection, even not directly involving central nervous system, can increase iNOS mRNA expression and NO production, which would induce death of neurons in hippocampus, resulting in impaired memory and deterioration of degenerative diseases such as Alzheimer's disease. Upregulated iNOS mRNA expression in the anterior pituitary and pineal gland causes large amounts of NO production, leading to reduced capability in anti-infection and responses to stress, decreased production of melatonin, and thus brain aging [24]. eNOS can inhibit aging-associated vascular senescence and dilated blood vessels that exert vascular protective effects. It has been reported that eNOS expression in endothelial cells is significantly reduced in aged mice [25]. Under physiological conditions, the central nervous system accurately controls NO production, release, distribution, and degradation by regulating the activation and inactivation of nNOS activity. In addition to its effect on the central nervous system, nNOS present in skeletal, cardiac, and smooth muscles plays an important role in regulating blood flow and muscular contraction [26]. Decreased nNOS expression has been shown to cause ischemic brain damage, early onset of senile dementia, Parkinson's disease, and other neurodegenerative diseases [27]. In the current study, we found that PSLKDT could impact NO, nNOS, eNOS, and iNOS to maintain homeostasis, thus preventing oxidative aging-associated dysregulation of these NO metabolism-related molecules.

SOD is a family of metalloenzymes that widely occur in organisms and serve as the key defense mechanism against damage caused by reactive oxygen species. Based on the difference in metal cofactors, there are three types of SODs: Cu/Zn-SOD, Mn-SOD, and Fe-SOD. Cu/Zn-SOD is a eukaryote enzyme that is primarily present in the plasma and chloroplast matrix of eukaryotic cells, and it is also found in animal blood and organs. Mn-SOD is primarily present in prokaryotic cells, eukaryotic cells, and in the mitochondrial matrix. Fe-SOD is primarily present in prokaryotic cells and some plants [28]. SOD has been shown to prevent and treat diseases that are related to superoxide free radicals. When superoxide anion free radicals are overproduced or SOD levels are low, excess superoxide anions could cause oxidative aging [29]. After exposed to oxidative damage or during aging, the body has decreased Cu/Zn-SOD and Mn-SOD levels [30]. CAT is an antioxidant enzyme, and it is mainly present in red blood cells and microsomes in some other tissue cells and is also found in the mitochondria and plasma [29, 30]. The organisms constantly produce ROS during the normal oxidative respiration. As highly reactive molecules, ROS contain unpaired electrons, which can be eliminated by antioxidant enzymes CAT, SOD, and GSH-Px. As the first line of defense, SOD dismutates O_2_^−^ to H_2_O_2_, which can be further reduced to H_2_O by CAT, together with increased oxygen content in cells [31]. GSH is directly or indirectly involved in various cellular activities, and one of the key functions is to work together with other related metabolic enzymes to form a strong defense against oxidative stress. GSH reduction system is composed of *γ*-glutamate-cysteine ligase (GSH1), glutathione synthetase (GSH2), glutathione reductase (GR), glutathione peroxidase (GPx), and NADPH; the interaction of these factors in this system assures inhibition of oxidative stress thus retarding oxidative aging of the body [32]. MDA is a product of lipid peroxidation in the body, and its levels can be used to assess the degree of oxidative aging [33]. Our results in the current study demonstrate that PSLKDT could significantly increase SOD, CAT, GSH (GSH1; GSH2), and GSH-Px levels and decrease MDA levels, thus effectively preventing D-galactose-induced oxidative aging.

HO-1 is a stress protein; in addition to participating in hemoglobin metabolism, it is involved in anti-inflammatory and antioxidative processes and has protective effects on cardiovascular and nervous neurological systems. Studies have shown that the antioxidant activity of HO-1 is particular pronounced in cardiovascular diseases such as atherosclerosis, ischemic heart disease, and hypertension, as well as in neurodegenerative diseases such as Alzheimer's disease, Parkinson's disease, and cerebral ischemic injury [34]. Nrf2 is important in maintaining endothelial integrity, and decreased Nrf2 function in blood vessel endothelial cells in aged individuals is closely associated with impaired endothelial functions. When oxidative stress is increased in the body, Nrf2 is released, which in turn promotes transcription and translation of HO-1, SOD, and CAT, leading to increased ability to eliminate oxygen free radicals in the body [35]. Nrf2 can also regulate *γ*-GCS production. When large amounts of ROS are produced due to dysregulated redox, Nrf2 is activated to promote *γ*-GCS expression, which consequently increases GSH synthesis and activation, leading to antioxidant effects [36]. In cells under oxidative stress, Nrf2 is dissociated from Keapl and is activated. Activated Nrf2 can translocate to the nuclei and bind to AREs to upregulate downstream genes for antioxidant enzyme NQO1, resulting in enhanced resistance to oxidative stress. Thus, the regulation of Nrf2/NQO1 signaling pathway is viewed as an efficient mechanism by which antioxidant molecules exert their antioxidant effects [37]. In the current study, we found that PSLKDT upregulated Nrf2 expression to further increase HO-1, *γ*-GCS, and NQO1 expression and thus prevented oxidative stress-induced aging.

Phenolic compounds contain active hydroxyl hydrogen, which reacts with free radicals to generate inert products or more stable free radicals, thus interrupting or delaying free radical-involved chain reactions. Catechins are natural antioxidants because these contain active hydroxyl groups that can eliminate free radicals. Their antioxidant activity is as potent as vitamins E and C. In addition to their free radical-scavenging effect, these are capable of precipitating protein and chelating several metal elements, which afford them inhibitory activity on certain oxidases, thus indirectly reducing free radicals in the body [38]. Pyrocatechol hydroxyl groups at the benzene ring of caffeic acid are the sites for antioxidative, free radical-scavenging activities; phenolic compounds reacting with free radicals lose hydrogen to form a stable semiquinone product and thus exert antioxidant effects [39]. EGCG is a natural compound with well-recognized antitumor properties, and its antioxidant activity is higher than vitamin C by 100-fold [40]. Studies have indicated that GCG has higher antioxidant activity than EGCG; EGCG and GCG have similar electronic effects; when exposed to highly active free radicals or high-energy singlet oxygen, the difference in antioxidant activity between GCG and EGCG depends on the difference in stereoconformation [41]. Thus, GCG has higher antioxidant activity than vitamin C. Ferulic acid can protect plasma membrane from free radical damage by scavenging free radicals, regulating physiological functions, inhibiting free radical-producing enzymes, promoting activities of free radical-scavenging enzymes such as GST and NAPPH, inhibiting tyrosinase activity, and binding to phosphatidylethanolamine [42]. Chlorogenic acid is an effective phenolic antioxidant. It contains R-OH groups that can form antioxidant hydrogen radicals to reduce the activity of hydroxyl and superoxide anion free radicals. Chlorogenic acid can protect tissues from oxidative damage, and its antioxidant activity is higher than caffeic acid and ferulic acid [43]. Isochlorogenic acid B contains more phenolic hydroxyl groups and has higher antioxidant activity compared to chlorogenic acid [44]. It is also reported that isochlorogenic acid and isochlorogenic acid A have higher* in vitro* antioxidant activity than vitamin C [45]. Several components present in PSLKDT have strong antioxidant activity, and this was proven in the current study, in that PSLKDT showed higher antioxidant activity than vitamin C, and these antioxidant compounds may contribute to the preventive effect of PSLKDT on oxidative aging in animals.

The results of the current study indicate that PSLKDT effectively prevents D-galactose-induced oxidative aging as evidenced by normalized parameters in serum, skin, liver, and spleen, and this effect is more potent than the well-recognized antioxidant vitamin C. HPLC analysis also confirms the presence of six potent antioxidant compounds, which alone or in combination exerts highly intense antioxidant effects, resulting in the prevention of aging in mice.* In vivo* animal studies have proven that PSLKDT is a promising form of polyphenol that is worth further investigation. Because Kuding tea is rich in PSLKDT, it is expected that Kuding tea may have potential applications as a polyphenol-rich health product or traditional Chinese medicine. While the study has made preliminary efforts in elucidating the mechanism of action of PSLKDT, further investigations, including those involving human subjects, are warranted to improve our understanding of this potential health product.

## Figures and Tables

**Table 1:** Gradient elution procedure.

No.	Time (min)	Current speed (mL/min)	0.1% formic acid (%)	Acetonitrile (%)
1	0	0.6	90	10
2	6.5	0.6	81.5	18.5
3	27.5	0.6	64.5	35.5
4	32.5	0.6	0	100
5	42.5	0.6	0	100

**Table 2:** Sequences of primers used in this study.

Gene Name	Sequence
nNOS	Forward: 5′-ACGGCAAACTGCACAAAGC-3′
	Reverse: 5′-CGTTCTCTGAATACGGGTTGTTG-3′

eNOS	Forward: 5′-TCAGCCATCACAGTGTTCCC-3′
	Reverse: 5′-ATAGCCCGCATAGCGTATCAG-3′

iNOS	Forward: 5′-GTTCTCAGCCCAACAATACAAGA-3′
	Reverse: 5′-GTGGACGGGTCGATGTCAC-3′

Cu/Zn-SOD	Forward: 5′-AACCAGTTGTGTTGTCAGGAC-3′
	Reverse: 5′-CCACCATGTTTCTTAGAGTGAGG-3′

Mn-SOD	Forward: 5′-CAGACCTGCCTTACGACTATGG-3′
	Reverse: 5′-CTCGGTGGCGTTGAGATTGTT-3′

CAT	Forward: 5′- GGAGGCGGGAACCCAATAG -3′
	Reverse: 5′- GTGTGCCATCTCGTCAGTGAA-3′

HO-1	Forward: 5′-ACAGATGGCGTCACTTCG-3′
	Reverse: 5′-TGAGGACCCACTGGAGGA-3′

Nrf2	Forward: 5′-CAGTGCTCCTATGCGTGAA-3′
	Reverse: 5′-GCGGCTTGAATGTTTGTC-3′

*γ*-GCS	Forward: 5′-GCACATCTACCACGCAGTCA-3′
	Reverse: 5′-CAGAGTCTCAAGAACATCGCC-3′

NQO1	Forward: 5′-CTTTAGGGTCGTCTTGGC-3′
	Reverse: 5′-CAATCAGGGCTCTTCTCG-3′

GAPDH	Forward: 5′-AGGTCGGTGTGAACGGATTTG-3′
	Reverse: 5′-GGGGTCGTTGATGGCAACA-3′

nNOS: neuronal nitric oxide synthase; eNOS: endothelial nitric oxide synthase; iNOS: inducible nitric oxide synthase; Cu/Zn-SOD: cuprozinc-superoxide dismutase; Mn-SOD: manganese superoxide dismutase; CAT: catalase; HO-1: heme oxygenase-1; Nrf2: nuclear factor-erythroid 2 related factor 2; *γ*-GCS: *γ*-glutamylcysteine synthetase; NQO1: NAD(P)H dehydrogenase [quinone] 1; GAPDH: glyceraldehyde-3-phosphate dehydrogenase.

**Table 3:** Contents of polyphenols of small-leaved Kuding tea.

Polyphenol	Catechin	Caffeic acid	GCG	Ferulic acid	Isochlorogenic acid B	Isochlorogenic acid A
Content (mg/g)	13.29	13.62	16.02	24.03	162.63	350.28

**Table 4:** Organ index of mice in each group.

Group	Thymus index	Brain index	Cardiac index	Liver index	Spleen index	Kidney index
Normal	0.26±0.03 ^a^	5.02±0.05 ^a^	3.69±0.05 ^a^	24.33±0.41 ^a^	1.67±0.05 ^a^	4.72±0.06 ^a^
Model	0.11±0.03 ^e^	3.18±0.03 ^e^	2.63±0.06 ^e^	16.18±0.33 ^e^	1.03±0.03 ^e^	3.02±0.05 ^e^
Vc	0.19±0.02 ^c^	4.66±0.07 ^c^	3.08±0.04 ^c^	20.08±0.32 ^c^	1.38±0.05 ^c^	4.09±0.07 ^c^
PSLKDT-L	0.15±0.02 ^d^	3.82±0.05 ^d^	2.79±0.06 ^d^	18.85±0.29 ^d^	1.22±0.06 ^d^	3.57±0.04 ^d^
PSLKDT-H	0.22±0.01 ^b^	4.81±0.04 ^b^	3.47±0.05 ^b^	22.82±0.36 ^b^	1.53±0.04 ^b^	4.51±0.03 ^b^

Values presented are the mean ± standard deviation (N=10/group).  ^a-d^Mean values with different letters over the same column are significantly different (*p*<0.05) according to Duncan's multiple range test. Vc: mice treated with vitamin C (100 mg/kg); PSLKDT-L: mice treated with low concentration of polyphenol of small-leaved Kuding tea (50 mg/kg); PSLKDT-H: mice treated with high concentration of polyphenol of small-leaved Kuding tea (100 mg/kg).

**Table 5:** The levels of NO, SOD, GSH-Px, GSH, and MDA in serum of mice.

Group	NO (*μ*mol/L)	SOD (U/mL)	GSH-Px (U/mL)	GSH (mg/L)	MDA (nmol/mL)
Normal	19.37±0.28 ^e^	235.18±12.37 ^a^	203.08±8.32 ^a^	45.01±0.42 ^a^	4.13±0.27 ^e^
Model	57.12±0.62 ^a^	67.85±5.88 ^e^	73.25±6.21 ^e^	10.85±0.51 ^e^	33.18±0.52 ^a^
Vc	36.18±0.50 ^c^	163.08±9.33 ^c^	131.09±8.39 ^c^	28.01±0.36 ^c^	12.54±0.43 ^c^
PSLKDT-L	47.19±0.44 ^b^	101.85±8.32 ^d^	108.37±6.53 ^d^	17.38±0.45 ^d^	21.08±0.30 ^b^
PSLKDT-H	24.10±0.36 ^d^	192.58±8.99 ^b^	172.26±6.08 ^b^	36.71±0.29 ^b^	7.18±0.41 ^d^

Values presented are the mean ± standard deviation (N=10/group). ^a-e^ Mean values with different letters over the same column are significantly different (*p*<0.05) according to Duncan's multiple range test. Vc: mice treated with vitamin C (100 mg/kg); PSLKDT-L: mice treated with low concentration of polyphenol of small-leaved Kuding tea (50 mg/kg); PSLKDT-H: mice treated with high concentration of polyphenol of small-leaved Kuding tea (100 mg/kg).

**Table 6:** The levels of NO, SOD, GSH-Px, GSH, and MDA in liver of mice.

Group	NO (*μ*mol/gprot)	SOD (U/mgprot)	GSH-Px (U/mgprot)	GSH (mg/ gprot)	MDA (nmol/mgprot)
Normal	2.38±0.18 ^e^	91.79±6.12 ^a^	181.94±8.49 ^a^	9.83±0.49 ^a^	1.79±0.16 ^e^
Model	8.37±0.26 ^a^	22.57±2.30 ^e^	73.08±5.27 ^e^	2.10±0.22 ^e^	8.93±0.29 ^a^
Vc	4.83±0.29 ^c^	69.48±5.32 ^c^	131.06±4.82 ^c^	6.79±0.34 ^c^	4.15±0.23 ^c^
PSLKDT-L	6.19±0.33 ^b^	49.79±5.61 ^d^	108.91±5.01 ^d^	4.83±0.28 ^d^	6.12±0.20 ^b^
PSLKDT-H	3.09±0.22 ^d^	80.43±5.83 ^b^	155.87±4.92 ^b^	7.91±0.32 ^b^	2.47±0.24 ^d^

Values presented are the mean ± standard deviation (N=10/group).  ^a-e^Mean values with different letters over the same column are significantly different (*p*<0.05) according to Duncan's multiple range test. Vc: mice treated with vitamin C (100 mg/kg); PSLKDT-L: mice treated with low concentration of polyphenol of small-leaved Kuding tea (50 mg/kg); PSLKDT-H: mice treated with high concentration of polyphenol of small-leaved Kuding tea (100 mg/kg).

**Table 7:** The levels of NO, SOD, GSH-Px, GSH, and MDA in spleen of mice.

Group	NO (*μ*mol/gprot)	SOD (U/mgprot)	GSH-Px (U/mgprot)	GSH (mg/ gprot)	MDA (nmol/mgprot)
Normal	1.63±0.12 ^e^	83.47±6.86 ^a^	130.91±7.83 ^a^	7.07±0.21 ^a^	0.87±0.12 ^e^
Model	9.32±0.19 ^a^	24.18±3.28 ^e^	34.18±4.25 ^e^	1.97±0.18 ^e^	5.12±0.36 ^a^
Vc	4.11±0.36 ^c^	58.71±4.83 ^c^	79.36±4.83 ^c^	4.02±0.22 ^c^	2.76±0.19 ^c^
PSLKDT-L	7.02±0.28 ^b^	39.82±5.19 ^d^	55.01±3.83 ^d^	2.83±0.16 ^d^	3.41±0.23 ^b^
PSLKDT-H	2.39±0.21 ^d^	67.89±4.87 ^b^	107.27±5.38 ^b^	5.93±0.24 ^b^	1.44±0.16 ^d^

Values presented are the mean ± standard deviation (N=10/group). ^a-e^ Mean values with different letters over the same column are significantly different (*p*<0.05) according to Duncan's multiple range test. Vc: mice treated with vitamin C (100 mg/kg); PSLKDT-L: mice treated with low concentration of polyphenol of small-leaved Kuding tea (50 mg/kg); PSLKDT-H: mice treated with high concentration of polyphenol of small-leaved Kuding tea (100 mg/kg).

## Data Availability

No data were used to support this study.

## References

[1] Che Y., Wang Z., Zhu Z. (2016). Simultaneous qualitation and quantitation of chlorogenic acids in Kuding tea using ultra-high-performance liquid chromatography-diode array detection coupled with linear ion trap-orbitrap mass spectrometer. *Molecules*.

[2] Lu Y., Zhou W., Feng Y. (2017). Acteoside and acyl-migrated acteoside, compounds in chinese kudingcha tea, inhibit *α*-amylase in vitro. *Journal of Medicinal Food*.

[3] Zhao X., Song J.-L., Yi R. (2018). Comparison of antioxidative effects of insect tea and its raw tea (kuding tea) polyphenols in kunming mice. *Molecules*.

[4] Zhao X., Sun P., Li G., Yi R., Qian Y., Park K.-Y. (2018). Polyphenols in Kuding tea help prevent HCl/ethanol-induced gastric injury in mice. *Food & Function*.

[5] Zhou Y. L., Feng X., Zhu K., Zhao X. (2015). Apoptosis inducing effects of crude polyphenols of Insect tea made by Kuding tea leaves in HepG2 human hepatoma cells. *Science Technology Food Industry*.

[6] Buford T. W. (2016). Hypertension and aging. *Ageing Research Reviews*.

[7] Chard S., Harris-Wallace B., Roth E. G. (2017). Successful aging among African American older adults with type 2 diabetes. *Journals of Gerontology - Series B Psychological Sciences and Social Sciences*.

[8] Kitada M., Ogura Y., Koya D. (2016). The protective role of Sirt1 in vascular tissue: its relationship to vascular aging and atherosclerosis. *AGING*.

[9] Rao K. S. (2009). Free radical induced oxidative damage to DNA: relation to brain aging and neurological disorders. *Indian Journal of Biochemistry and Biophysics*.

[10] Hohensinner P. J., Kaun C., Ebenbauer B. (2018). Reduction of premature aging markers after gastric bypass surgery in morbidly obese patients. *Obesity Surgery*.

[11] Li L., Xu M., Shen B., Li M., Gao Q., Wei S.-G. (2016). Moderate exercise prevents neurodegeneration in D-galactose-induced aging mice. *Neural Regeneration Research*.

[12] Li H. Y., Xia J. Q., Yang L. Y., Zhong R. Z. (2013). Plant polyphenols: antioxidant capacity and application in animal production. *Chinese Journal of Animal Nutrition*.

[13] Carluccio M. A., Siculella L., Ancora M. A. (2003). Olive oil and red wine antioxidant polyphenols inhibit endothelial activation: antiatherogenic properties of Mediterranean diet phytochemicals. *Arteriosclerosis, Thrombosis, and Vascular Biology*.

[14] Sharma S., Rana S., Patial V., Gupta M., Bhushan S., Padwad Y. S. (2016). Antioxidant and hepatoprotective effect of polyphenols from apple pomace extract via apoptosis inhibition and Nrf2 activation in mice. *Human & Experimental Toxicology*.

[15] Magro D. D.-D., Roecker R., Junges G. M. (2016). Protective effect of green tea extract against proline-induced oxidative damage in the rat kidney. *Biomedicine & Pharmacotherapy*.

[16] Pan Y., Long X., Yi R., Zhao X. (2018). Polyphenols in liubao tea can prevent CCl4-induced hepatic damage in mice through its antioxidant capacities. *Nutrients*.

[17] Qian Y., Zhang J., Zhou X. (2018). *Lactobacillus plantarum* CQPC11 isolated from sichuan pickled cabbages antagonizes d-galactose-induced oxidation and aging in mice. *Molecules*.

[18] Zhang J., Zhou X., Chen B. (2018). Preventive effect of *lactobacillus plantarum* CQPC10 on activated carbon induced constipation in institute of cancer research (ICR) mice. *Applied Sciences*.

[19] Li G., Wang J., Cheng Y. (2018). Prophylactic effects of polymethoxyflavone-rich orange peel Oil on N*ω*-nitro-l-arginine-induced hypertensive rats. *Applied Sciences*.

[20] Tang T., He B. (2013). Treatment of d-galactose induced mouse aging with *Lycium barbarum* polysaccharides and its mechanism study. *African Journal of Traditional, Complementary and Alternative medicines (AJTCAM)*.

[21] Khan S. S., Singer B. D., Vaughan D. E. (2017). Molecular and physiological manifestations and measurement of aging in humans. *Aging Cell*.

[22] Manini T. M. (2010). Energy expenditure and aging. *Ageing Research Reviews*.

[23] Tessari P. (2015). Nitric oxide in the normal kidney and in patients with diabetic nephropathy. *Journal of Nephrology*.

[24] Wells S. M., Holian A. (2007). Asymmetric dimethylarginine induces oxidative and nitrosative stress in murine lung epithelial cells. *American Journal of Respiratory Cell and Molecular Biology*.

[25] Fukai T., Siegfried M. R., Ushio-Fukai M., Cheng Y., Kojda G., Harrison D. G. (2000). Regulation of the vascular extracellular superoxide dismutase by nitric oxide and exercise training. *The Journal of Clinical Investigation*.

[26] Bonthius Jr. D. J., Winters Z., Karacay B., Bousquet S. L., Bonthius D. J. (2015). Importance of genetics in fetal alcohol effects: Null mutation of the nNOS gene worsens alcohol-induced cerebellar neuronal losses and behavioral deficits. *NeuroToxicology*.

[27] Lee M. H., Hyun D.-H., Jenner P., Halliwell B. (2001). Effect of proteasome inhibition on cellular oxidative damage, antioxidant defences and nitric oxide production. *Journal of Neurochemistry*.

[28] Kosenko E. A., Tikhonova L. A., Alilova G. A. (2017). Portacaval shunting causes differential mitochondrial superoxide production in brain regions. *Free Radical Biology & Medicine*.

[29] Selvaratnam J. S., Robaire B. (2016). Effects of aging and oxidative stress on spermatozoa of superoxide-dismutase 1- and catalase-null mice. *Biology of Reproduction*.

[30] Pawlak W., Kedziora J., Zolynski K. (1998). Effect of long term bed rest in men on enzymatic antioxidative defence and lipid peroxidation in erythrocytes. *Journal of Gravitational Physiology*.

[31] Berndt C., Lillig C. H. (2017). Glutathione, glutaredoxins, and iron. *Antioxidants & Redox Signaling*.

[32] Vázquez-Medina J. P., Zenteno-Savín T., Forman H. J., Crocker D. E., Ortiz R. M. (2011). Prolonged fasting increases glutathione biosynthesis in postweaned northern elephant seals. *Journal of Experimental Biology*.

[33] Hosen M. B., Islam M. R., Begum F., Kabir Y., Howlader M. Z. H. (2015). Oxidative stress induced sperm DNA damage, a possible reason for male infertility. *Iranian Journal of Reproductive Medicine*.

[34] Sue Y.-M., Cheng C.-F., Chang C.-C., Chou Y., Chen C.-H., Juan S.-H. (2009). Antioxidation and anti-inflammation by haem oxygenase-1 contribute to protection by tetramethylpyrazine against gentamicin-induced apoptosis in murine renal tubular cells. *Nephrology Dialysis Transplantation *.

[35] Hong C., Cao J., Wu C.-F. (2017). The Chinese herbal formula Free and Easy Wanderer ameliorates oxidative stress through KEAP1-NRF2/HO-1 pathway. *Scientific Reports*.

[36] Iwayama K., Kusakabe A., Ohtsu K. (2017). Long-term treatment of clarithromycin at a low concentration improves hydrogen peroxide-induced oxidant/antioxidant imbalance in human small airway epithelial cells by increasing Nrf2 mRNA expression. *BMC Pharmacology & Toxicology*.

[37] Jiang X.-P., Tang J.-Y., Xu Z. (2017). Sulforaphane attenuates di-N-butylphthalate-induced reproductive damage in pubertal mice: Involvement of the Nrf2-antioxidant system. *Environmental Toxicology*.

[38] Wang S. X., Li A. M., Zhang J. J., Ou S. Y., Huang X. S., Zhang G. W. (2011). HPLC-ESI-MS analysis of phenolic compounds in different solvent fractions of ethanol extract of longan seeds and their antioxidant activities. *Food Science*.

[39] Bao Y. F., Shen X. C., Wang F. (2018). The research progress and development prospects of biosynthesis, structure activity relationship and biological activity of caffeic acid and its main types of derivatives. *Natural Product Research and Development*.

[40] Yilixiati X., Li X.-J. (2012). Inhibitory effect of epigallocatechin gallate on the proliferation of Lewis Lung Cancer mice and A549 and Calu-3 lung cancer cells. *Chinese Pharmaceutical Journal*.

[41] Shen S. R., Jin C. F., Yang X. Q., Zhao B. L. (2000). Study on the scavenging effects of EGCG and GCG on singlet oxygen with ESR method. *Journal Tea Science*.

[42] Ou S. Y. (2002). The function and application of ferulic acid. *Guangzhou Food Science Technology*.

[43] Bouayed J., Rammal H., Dicko A., Younos C., Soulimani R. (2007). Chlorogenic acid, a polyphenol from Prunus domestica (Mirabelle), with coupled anxiolytic and antioxidant effects. *Journal of the Neurological Sciences*.

[44] Liu X., Xu X. W. (2017). Protective effect of isochlorogenic acid B on CCl_4_ induced acute liver injury in mice. *Journal of International Pharmaceutical Research*.

[45] Hou C. P., Han L. W., Zhang F. (2017). Study on the antioxidant activity of isochlorogenic acid A. *Science Technology Food Industry*.

